# Strategies for p53 Activation and Targeted Inhibitors of the p53-Mdm2/MdmX Interaction

**DOI:** 10.3390/cells14080583

**Published:** 2025-04-12

**Authors:** Ye Huang, Wang Li, Yuke Zhou, Jinping Bai, Ning Li, Zhengding Su, Xiyao Cheng

**Affiliations:** 1 Institute of Modern Fermentation Engineering and Future Foods, School of Light Industry and Food Engineering, Guangxi University, No. 100, Daxuedong Road, Nanning 530004, China; 2216392005@st.gxu.edu.cn (Y.H.); 2416393006@st.gxu.edu.cn (W.L.); 2405120112@st.gxu.edu.cn (Y.Z.); 2316392001@st.gxu.edu.cn (J.B.); ningli2023@gxu.edu.cn (N.L.); 2 School of Pharmaceutical Sciences and Institute of Materia Medica, Xinjiang University, Urumqi 830017, China; james_su@xju.edu.cn

**Keywords:** cancer, p53, inhibitors, Mdm2, MdmX

## Abstract

*p53* is a tumor suppressor gene and is regarded as one of the most crucial genes in protecting humans against cancer. The protein Mdm2 and its homolog MdmX serve as negative regulators of p53. In nearly half of cancer cells, there is an overexpression of Mdm2 and MdmX, which inhibit p53 activity. Furthermore, Mdm2’s E3 ubiquitin ligase activity promotes the ubiquitination and degradation of p53. Therefore, blocking the interaction between p53 and Mdm2/MdmX to prevent the degradation of wild-type p53 is an effective strategy for inhibiting tumor growth. This paper primarily discusses the regulatory relationship between p53, MdmX and Mdm2, and provides a review of the current status of p53-Mdm2/MdmX inhibitors. It aims to offer a theoretical foundation and research direction for the future discovery and design of targeted inhibitors against the p53-Mdm2/MdmX interaction.

## 1. Introduction

**Figure 1:**
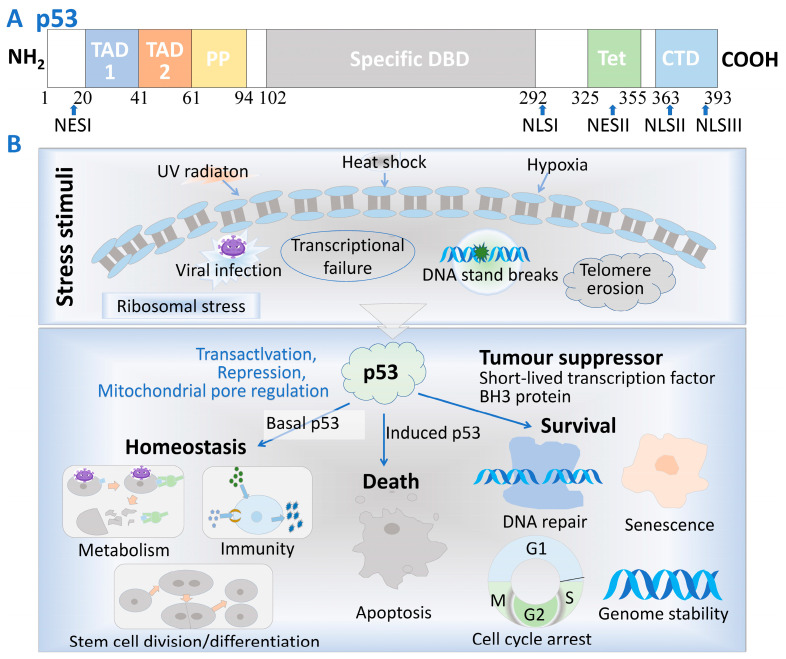
The structure of human p53 and its physiological functions. (**A**) The N-terminal region of p53 (residues 1–101) contains two transcription activation domains, TAD1 and TAD2, along with a proline-rich region PP (residues 61–94), featuring five PXXP motifs, which are crucial for its apoptotic function. Additionally, a nuclear export signal (NES) is located between residues 11 and 27. The central core domain (amino acids 102–292) includes the sequence-specific DNA binding domain (DBD). Most missense mutations in the p53 gene occur within this domain, which harbors a highly conserved subregion. The C-terminal region of p53 (residues 292–393) consists of a flexible linker (residues 292–324) that connects the core domain to the tetramerization domain (Tet, residues 325–355) and the C-terminal regulatory domain (CTD, residues 363–393). The C-terminal region also contains both NES and nuclear localization signal (NLS) sequences. (**B**) In response to stimuli such as DNA damage, hypoxia, and ribosomal stress, the p53 pathway is activated in cells. In cases of moderate damage, cancer cells repair through p21 mediation, while severe damage triggers the activation of apoptotic genes. In normal cells, the pathway primarily supports repair processes and plays a crucial role throughout various phases of the cell lifecycle, including cell division, differentiation, metabolism, and more.

The structure of the tumor suppressor gene *p53* is depicted in Figure 1A. It comprises two adjacent transcription activation domains (TADs), referred to as TAD1 and TAD2, located at the N-terminus. TAD2 overlaps with a proline-rich domain, which is vital for apoptosis and the stabilization of p53 function [1]. The central or “core” domain contains the sequence-specific DNA-binding region of p53, which is crucial for the transcriptional activation of certain genes and tumor suppression. The C-terminal region includes sequences required for nuclear localization, non-specific DNA binding, and regulatory functions [2,3].

When cells are exposed to damage from factors such as DNA damage or ribosomal stress, as shown in Figure 1B, the p53 pathway is activated to repair the damage. If the DNA damage is severe, p53 can initiate cell apoptosis. p53 is essential for a variety of biological processes, including DNA repair, cell cycle arrest, metabolism, aging, and apoptosis [4,5]. Abnormalities in p53 are commonly observed in tumors, primarily involving p53 mutations (mutp53) or functional inactivation of wild-type p53 (wtp53) [6]. Mutations in the *p53* gene (*TP53*) impair the tumor-suppressive activity of the p53 protein it encodes, with nearly half of all tumors carrying mutated p53. Most *TP53* mutations are single missense mutations within its functional domains, which result in the loss of wtp53 function. Consequently, there has been growing interest in developing small molecules that restore wild-type-like function to mutant p53 proteins.

## 2. The Regulatory Circuit Between p53 and Mdm2/MdmX

**Figure 2:**
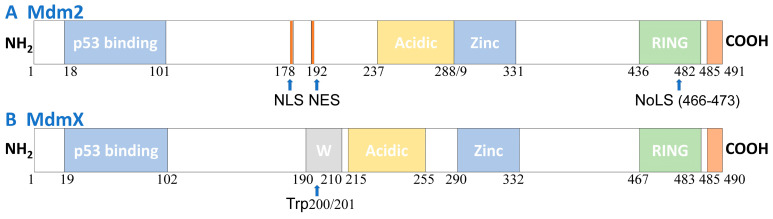
The structures of Mdm2 and MdmX. (**A**) The Mdm2 structure includes the N-terminal p53 binding domain (residues 18–101), a central acidic domain (residues 237–288). It also contains a zinc finger motif (residues 289–331) involved in ribosomal protein interactions. The C-terminal region of Mdm2 contains a RING (Really Interesting New Gene) domain (residues 436–482) with E3 ubiquitin ligase activity, while the C-terminal tail (residues 485–491) regulates the RING motif by promoting Mdm2 homodimer formation and Mdm2-MdmX heterodimerization. (**B**) MdmX shares structural similarities with Mdm2, containing an N-terminal p53 binding domain (residues 19–102), a central acidic region (residues 215–255), a zinc finger region (residues 290–332), and a C-terminal RING domain (residues 467–483). MdmX lacks NLS, NES, and nucleolar localization signal (NoLS) sequences but features a unique WWW motif (W, residues 190–210) that inhibits its interaction with p53. In contrast to Mdm2, the RING domain of MdmX does not exhibit E3 ligase activity. Abbreviations: C′, C-terminal; F, phenylalanine; L, leucine; N′, N-terminal; W, tryptophan.

Mdm2 (Figure 2A) and its homolog MdmX (Figure 2B) are negative regulatory factors of p53, primarily regulating the stability and activity of the p53 protein. The Mdm2-p53 interaction involves three critical regions of these proteins: (1) The main interaction site lies within the N-terminal domain of Mdm2 and the TAD1 region of p53. This interaction prevents p53 from binding to the transcriptional activation domains of its target genes; (2) The central region of Mdm2 (the acidic domain) interacts with the sequence-specific DNA binding domain (DBD) of p53, a key step for p53 ubiquitination [7,8]; (3) The N-terminal domain of Mdm2 also interacts with the C-terminal domain of p53, a region necessary for post-translational modifications such as phosphorylation and ubiquitination. Among them, ubiquitination also requires the involvement of the RING domain [9]. The negative feedback regulation between Mdm2 and p53 occurs primarily through two mechanisms: first, Mdm2 directly binds to the N-terminal of p53, inhibiting its transcriptional activation function [10]. Second, Mdm2 functions as an E3 ubiquitin ligase, facilitating p53 degradation through ubiquitination [2,11].

The structure of MdmX, a homolog of Mdm2, is shown in Figure 2B. MdmX lacks E3 ligase activity in its RING domain and does not directly induce p53 degradation. However, it interacts with Mdm2 through its RING motif, and this interaction regulates the levels of both p53 and Mdm2 [12].

**Figure 3:**
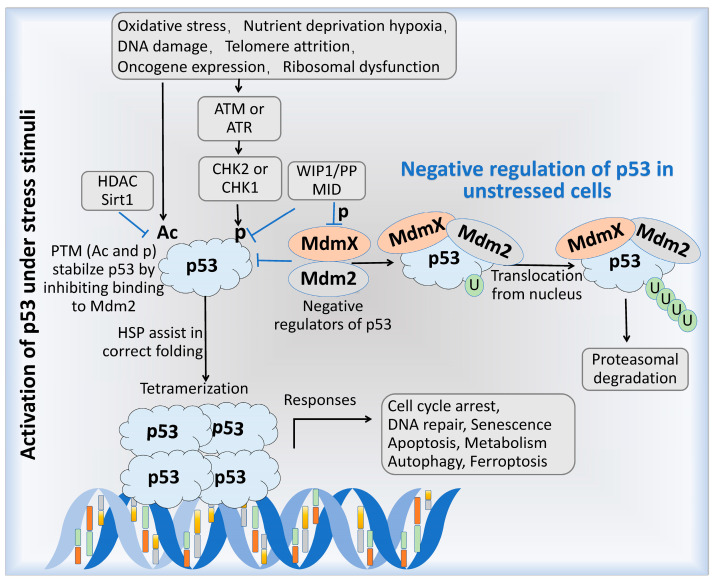
The regulatory loop between p53 and Mdm2/MdmX. Under non-stress conditions, p53 levels are maintained at low levels primarily through binding to its key negative regulators, Mdm2 and MdmX. In response to stress stimuli, post-translational modifications (PTMs), such as acetylation (Ac) or phosphorylation (p) of checkpoint kinases CHK1 and CHK2, are induced by the activation of upstream kinases, Ataxia Telangiectasia Mutated (ATM) and Ataxia Telangiectasia and Rad3-related protein (ATR). These modifications stabilize and activate p53 by preventing its binding to Mdm2. Subsequently, heat shock proteins (HSPs) aid in the proper folding of p53 monomers, leading to the formation of its active tetrameric form, which binds to DNA for gene transcription. During this activation process, p53 inhibition can occur through the removal of PTMs: the histone deacetylase (HDAC) sirtuin 1 (SirT1) induces deacetylation of p53 and promotes its ubiquitination by Mdm2. Additionally, p53 induces the expression of another negative regulator, WIP1/PPM1D, a phosphatase 1, which destabilizes p53 by dephosphorylating serine 15 on p53 and dephosphorylating Mdm2, thereby promoting the stabilization of Mdm2.

Mdm2 and MdmX, as regulatory factors of the *p53* gene, primarily control the stability and activity of the p53 protein, thereby contributing to processes such as cell growth inhibition, apoptosis induction, and cell cycle regulation [4]. The interactions among these three proteins are regulated through a feedback loop [13], as illustrated in Figure 3. Under non-stress conditions, p53 levels remain low by binding to its primary negative regulators, Mdm2 and MdmX, which suppress its activity and promote its ubiquitination and degradation. Under stress conditions, post-translational modifications (PTMs) to p53, such as acetylation (Ac) [14,15] or phosphorylation (p) [16], inhibit the interaction between p53 and Mdm2, thereby stabilizing and activating p53. Heat shock proteins (HSPs) aid in the proper folding of p53 monomers, allowing the formation of an active tetrameric form [17,18], which then binds to DNA, participates in gene transcription, and plays a regulatory role at various stages of the cell cycle [19].

## 3. Strategies for Activating p53

In normal cells, p53 levels are maintained at low levels by its negative regulators, Mdm2 and MdmX. *TP53* mutations can impair p53 activity, necessitating distinct small-molecule activation strategies depending on the *TP53* status of the tumor [5,20]. For tumors with missense mutations in *TP53*, small molecule drug development primarily focuses on compounds that restore the wild-type conformation of mutp53. Conversely, for cancers with wtp53, the main research focus is on designing small molecules that release p53 from its negative regulators (Mdm2, MdmX), thereby reactivating its function [21].

### 3.1. Activate the Activity of mutp53

Among the approximately 22,000 genes in the human genome, *TP53* is the most frequently mutated gene in carcinogenesis. *TP53* mutations are found in about 50% of human cancers, leading to the loss of the tumor-suppressive function of the p53 protein, thereby promoting tumor initiation and progression. As a result, these mutants are considered promising targets for small molecules aimed at restoring the conformation and function of mutp53 [22,23] providing new opportunities for cancer treatment.

In 1999, Foster et al. [24] reported the first small molecule capable of reactivating mutp53, CP-31398. This compound was shown to stabilize the wild-type conformation of p53 under denaturing conditions, restoring its transactivation ability and antitumor activity in vivo. This breakthrough provided a novel approach for cancer therapies targeting mutp53. However, subsequent studies revealed that the mechanism of action of CP-31398 was more complex than originally anticipated. In addition to inserting into DNA and causing nonspecific toxicity, CP-31398 also promoted the p53-independent upregulation of the apoptosis protein BAX [25]. Due to these complex effects and nonspecific toxicity, CP-31398 was ultimately not pursued for clinical development. Nonetheless, its discovery laid a crucial foundation for developing mutp53-targeted cancer therapies and spurred the creation of additional small molecules aimed at reactivating mutp53. Since then, other mutp53 reactivation agents have emerged. Zache et al. [26] reported that the low molecular weight compound STIMA-1 can bind to mutant p53 DNA in vitro, stimulate the expression of p53 proteins, and trigger apoptosis in human tumor cells carrying mutp53. Another small molecule, PRIMA-1 [27,28] and its structural analog, PRIMA-1^MET^ (also known as APR-246) [27], reactivates mutp53 through the formation of adducts with the thiol groups of wtp53. This covalent modification induces conformational changes in mutp53, allowing the modified p53 to trigger apoptosis in tumor cells. APR-246 has been successfully tested in Phase I/II clinical trials for blood cancers and prostate malignancies [29,30]. Additionally, a Phase Ib/II clinical trial of APR-246 combined with azacytidine for treating p53 mutant myelodysplastic syndromes demonstrated good tolerability [31]. APR-246 is also being tested, in combination with other therapies, in several ongoing Phase I/II trials [32]. Certain missense mutations in p53 can lead to its self-aggregation, resulting in the formation of amyloid proteins. Soragni et al. [33] reported a cell-penetrating peptide called ReACp53, which can inhibit the formation of p53 amyloid aggregates. This action restores the tumor-suppressive function of p53, reduces tumor proliferation in high-grade serous ovarian carcinomas (HGSOC), and shrinks xenografts.

In addition to reactivating mutp53, selectively targeting the mutp53 protein may also exert antitumor effects [34]. The development strategy for this compound is founded on two key observations: (1) silencing mutp53 through siRNA or shRNA can effectively inhibit mutp53-driven malignant progression, and (2) mutp53 exhibits intrinsic instability [35,36,37]. Therefore, the cornerstone of this strategy lies in limiting the expression of mutp53 and promoting its degradation. Several types of compounds have been developed based on this approach for the treatment of mutp53-related cancers, including:

(a) Hsp90 inhibitors: These inhibitors prevent the binding of Hsp90 chaperones to mutp53, thereby facilitating its degradation [38];
(b) HDAC inhibitors: These agents suppress transcriptional activity regulated by HDAC and disrupt the HDAC6/Hsp90/mutp53 complex [39];
(c) Statins: Statins inhibit the interaction between mutp53 and DNAJA1, inducing CHIP-dependent mutp53 degradation [40];
(d) Gambogic acid: This compound enhances wtp53 levels, disrupts the mutp53/Hsp90 complex, and promotes CHIP-mediated degradation of mutp53 [41];
(e) Spautin-1: Spautin-1 inhibits macro autophagy and induces mutp53 degradation through partner-mediated autophagy [42].

These compounds represent promising therapeutic strategies targeting mutp53-driven malignancies.

Scientists are actively working to bring new breakthroughs in cancer treatment by developing small-molecule drugs that restore the function of mutp53. These efforts hold the potential to improve survival rates and quality of life for cancer patients, while also advancing the field of cancer therapy.

### 3.2. Unleash the Tumor-Suppressive Activity of wtp53

In cancers with wtp53, current research is focused on inhibiting p53 degradation. The primary strategy involves blocking the interaction between p53 and the E3 ubiquitin ligase Mdm2 [43,44], along with screening and designing small molecules that target the p53-Mdm2 binding site to restore p53 activity [45]. Additionally, some cancers show increased expression of MdmX, a homolog of Mdm2, which can directly bind to p53 and inhibit its transcriptional activity. Furthermore, when bound to Mdm2, MdmX enhances Mdm2’s E3 ligase activity, accelerating p53 ubiquitination and degradation [46,47,48]. This suggests that inhibiting the p53-MdmX interaction may offer therapeutic benefits, positioning MdmX as a novel target for cancer treatment. In nearly half of cancer cells, the overexpression of Mdm2 or MdmX proteins inhibits p53 activity, resulting in persistently low levels of p53 protein.

Therefore, for cancers with an overexpression of Mdm2/MdmX, developing effective inhibitors that target Mdm2/MdmX to release p53 and restore its normal tumor-suppressive function could provide new approaches to overcoming the challenges of cancer prevention and treatment.

#### 3.2.1. Inhibitors Targeting the p53-Mdm2 Interaction

**Figure 4:**
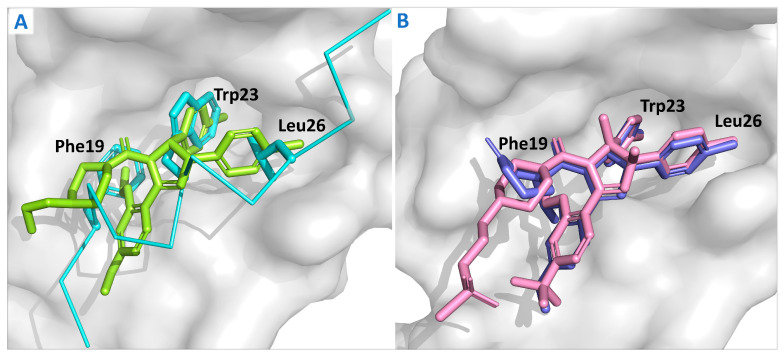
Comparison of the crystal structures of Mdm2 complexes with their different ligands. (**A**) Comparison of the binding modes of Nutlin-2 (green) and p53p (cyan) with Mdm2 proteins (PDB ID: 1RV1 and 1YCR); (**B**) Comparison of the binding modes between Nutlin-3a (purple) and RG7112 (pink) with Mdm2 proteins (PDB ID: 4J3E and 4IPF).

The crystal structure of the Mdm2 protein complex with the ligand Nutlin-2 (Figure 4A) offers the first detailed view of the precise binding interaction between Mdm2 and the small-molecule inhibitor, laying a solid theoretical foundation for the development of Mdm2 inhibitors. The structure shows that p53 binds tightly to a specific pocket on the Mdm2 surface through key amino acid residues, such as Phe19, Trp23, and Leu26 [48]. Based on this insight, a range of Mdm2 inhibitors with varied structures has been successfully developed [49], including compounds derived from quinazolinone, spirooxindole, pyrrolidone, piperidone, and pyrrole-hexene-imidazole [46,50,51]. These inhibitors effectively mimic the α-helix structure of p53 in solution or in vivo, occupying the binding pocket, inhibiting Mdm2 activity, and releasing p53, which ultimately restores or enhances p53’s tumor-suppressive function.

**Figure 5:**
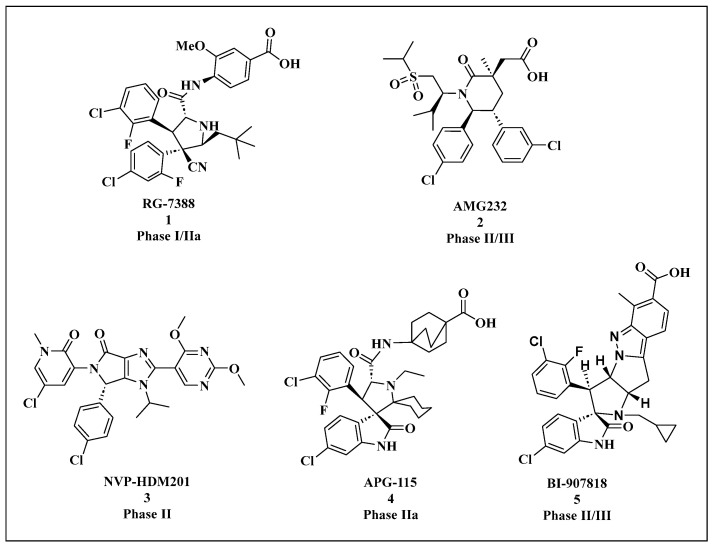
Chemical structures of Mdm2 inhibitors in clinical trials that have either started or are currently recruiting patients.

Most Mdm2 inhibitors have shown limited efficacy in Phase I trials, with significant thrombocytopenia emerging as a dose-limiting toxicity linked to prolonged Mdm2 inhibition (Table 1). Nonetheless, several small-molecule Mdm2 inhibitors are currently undergoing Phase II/III clinical trials for the treatment of WTp53 tumors, as shown in Figure 5.

In 2004, Vassilev et al. [46], introduced the first class of Mdm2 small-molecule inhibitors, known as Nutlins, which feature an imidazoline scaffold. Among them, Nutlin-3a was the most potent, with a binding affinity to Mdm2 of 90 nM. Nutlin-3a can target the p53-binding site on the Mdm2 protein (Phe19, Trp23, Leu26), blocking their interaction, reducing p53 degradation, and thereby inducing apoptosis in tumor cells [61]. Nutlin-3a is dependent on wtp53 and shows limited anti-proliferative activity in mutp53 tumor cells (MDA-MB-435 and SW480) [62]. The Nutlin-3a derivative RG7112 was the first Mdm2 inhibitor to enter clinical trials. These trials demonstrated that Mdm2 inhibition activates the p53 pathway and reduces cell proliferation in Mdm2-amplified liposarcoma. Specifically, on day 8, p53 and p21 levels increased by 4.86-fold and 3.48-fold, respectively, while Mdm2 mRNA expression increased by 3.03-fold [63,64]. The crystal structure is shown in Figure 4B. RG7112 demonstrated growth inhibition and cytotoxic effects on SJSA-1 osteosarcoma cells, which overexpress Mdm2 [65], in vitro. RG7112 has been tested clinically in chronic myelogenous leukemia (CML), acute myeloid leukemia (AML), solid tumors, and hematological malignancies. However, RG7112 showed poor tolerance at the required high doses and induced relatively severe hematological and gastrointestinal toxicities. As a result, RG7112 was later replaced by the third-generation derivative, RG-7388 (idasanutlin) (compound 1).

Rew Y et al. [66] designed and synthesized a series of hexagonal ring scaffolds, and through structural optimization, developed the oral Mdm2 inhibitor AMG232 (compound 2). Studies revealed that treatment with AMG232 in SJSA-1 cells resulted in a robust induction of p21 within 24 h, peaking at 30 times the baseline level four hours after administration. These data indicate that AMG232 effectively activates the p53 signaling pathway in human tumors in vivo. AMG232 enhances the tumor-suppressive function of wtp53 in osteosarcoma cells, resulting in tumor regression [67]. Furthermore, when combined with other cytotoxic cancer drugs, AMG232 demonstrates improved anti-tumor efficacy without significant side effects [68]. Research by Xiao Y et al. showed that AMG232 inhibits glioma angiogenesis by blocking the p53-Mdm2 interaction in the p53-RBM4-VEGFR2 pathway, thereby suppressing glioma endothelial cell proliferation [69]. In a Phase I trial by Gluck W L et al., AMG232 exhibited good safety and pharmacokinetic profiles in patients with advanced wtp53 solid tumors or multiple myeloma, showing anti-tumor activity [70]. A Phase I trial by Erba H P et al. in AML patients revealed that AMG232 treatment caused gastrointestinal-related adverse reactions, but the drug demonstrated acceptable pharmacokinetics, targeted effects, and anti-cancer activity, warranting further investigation in relapsed/refractory AML patients [54]. The most commonly reported AEs are gastrointestinal (such as diarrhea, nausea, vomiting) and hematologic (such as thrombocytopenia, anemia, neutropenia) [71].

HDM201 (compound 3) is an imidazopyrrolidone analog that demonstrates strong efficacy and favorable physicochemical properties. Both intermittent high-dose and continuous low-dose treatment regimens led to complete and sustained tumor regression in the SJSA-1 xenograft model and the rat HSAX2655 LPS PDX model. Furthermore, HDM201 treatment resulted in the upregulation of p21 and PUMA expression, indicating robust activation of the p53 pathway [72]. In multiple clinical trials, HDM201 has shown a manageable safety profile. Consistent with other Mdm2 inhibitors, delayed thrombocytopenia, neutropenia, anemia, and gastrointestinal side effects were observed in patients with hematologic malignancies, though these effects were not seen in solid tumor patients, suggesting a treatment-related cause [73,74]. HDM201 is currently being studied or planned for use in early-phase clinical trials (Phase I and I/II) in combination with other drugs, with the goal of enhancing its therapeutic efficacy.

Wang et al. [75] developed the small-molecule inhibitor MI-77301 (SAR405838) by targeting the p53-Mdm2 protein–protein interaction (PPI). This compound was derived by substituting groups exposed to the solvent region of MI-888, achieving the binding affinity of 0.88 nM with Mdm2, demonstrating high selectivity and specificity compared to other proteins. MI-77301 effectively activates wtp53 both in vitro and in xenograft tumor models of leukemia and solid tumors, leading to p53-dependent cell cycle arrest and apoptosis. Phase I clinical trials in patients with advanced solid tumors indicated that MI-77301 has favorable safety and pharmacokinetic profiles, although it could not be administered at the planned maximum tolerated dose on a weekly basis [76]. The compound was subsequently optimized, resulting in the development of APG-115 (Compound 4), which overcomes the limitations of MI-77301, such as slow isomerization reactions and poor solubility in neutral environments [77]. APG-115 demonstrated high affinity for Mdm2 (K_i_ < 1 nM) and effectively inhibited cell growth in acute leukemia (RS411), prostate cancer (LNCaP), and colon cancer (HCT116) cell lines, with IC_50_ values of 38 nM, 18 nM, and 104 nM, respectively. Additionally, it showed favorable oral pharmacokinetic properties. In the SJSA-1 tumor model, APG-115 strongly activated p53, elevating levels of p53, Mdm2, ubiquitinated Mdm2 (ub-Mdm2), and p21, inducing robust apoptosis. Studies by Yi H et al. [78] demonstrated that APG-115 activates wtp53 and enhances the anti-tumor effects of radiotherapy in gastric cancer, both in vitro and in vivo. Zhai Y et al. investigated the combination of APG-2575 and APG-115 in treating acute myeloid leukemia (AML) and acute lymphoblastic leukemia xenograft models. The results showed that the combination exhibited synergistic anti-proliferative and pro-apoptotic effects in wtp53 AML cell lines, overcoming resistance to APG-2575 [79]. The Mdm2 inhibitor BI-0252 [80], developed by Gollner et al., demonstrates an Mdm2 binding affinity with an IC_50_ of 4 nM. BI-907828 [81] (compound 5), a derivative optimized from BI-0252, exhibits high permeability, good bioavailability, and a low effective dose, making it suitable for intermittent oral dosing regimens [82]. BI-907828 effectively induces the upregulation of the p53 transcriptional targets p21 and PUMA. It demonstrated tumor regression in the SJSA-1 osteosarcoma xenograft model and a mouse model of dedifferentiated liposarcoma (DDLPS) [80,83,84]. BI-907828 has also shown preliminary efficacy in a Phase I trial involving patients with advanced or metastatic solid tumors [82]. Ongoing Phase I clinical trials are exploring its use in advanced or metastatic solid tumors. Additionally, BI-907828 is being tested as a monotherapy in Phase IIa/IIb trials for locally advanced or metastatic Mdm2-overexpressing wtp53 biliary adenocarcinoma, pancreatic ductal adenocarcinoma, urothelial bladder cancer, and lung adenocarcinoma, with participant recruitment currently in progress.

Although clinical trials have shown that Mdm2 inhibitors possess anti-tumor activity and an acceptable safety profile, they come with limitations, including drug resistance and dose-limiting toxicities. Common resistance mechanisms include p53 mutations in wtp53 tumors, alterations in various oncogenic pathways, and the overexpression of MdmX, among others. For instance, Michaelis et al. [85] conducted continuous nutlin-3 treatment on various p53 wild-type cancer cell lines and observed the emergence of new p53 mutations at different sites, which were absent in the original cell population. Similarly, Milademetan also induces p53 mutations. In the Phase Ib/II clinical trial of Milademetan, Koyama et al. [86] found that acquired *TP53* mutations were consistently detected in the blood of patients with intimal sarcoma. Chapeau et al. [87] administered HDM201 to xenograft models and discovered that, among the most frequently acquired drug-resistant tumors, 54% exhibited transformation-related p53 mutations that resulted in functional loss. Furthermore, p63 and p73 were shown to produce transposon-mediated dominant-negative N-terminal truncations. In addition, the expression levels of B-cell lymphoma-extra-large (Bcl-xL) and MdmX proteins were significantly upregulated in tumor cells. Additionally, the upregulation of p73 expression in the p53-Mdm2 feedback loop has been shown to contribute to the development of drug resistance in cancer cells [88].

These challenges have sparked interest in developing MdmX inhibitors and Mdm2/MdmX dual inhibitors to address the shortcomings of current Mdm2 inhibitors and improve therapeutic outcomes for related cancers.

#### 3.2.2. Monotherapy p53-MdmX Inhibitors and Dual p53-Mdm2/MdmX Inhibitors

**Figure 6:**
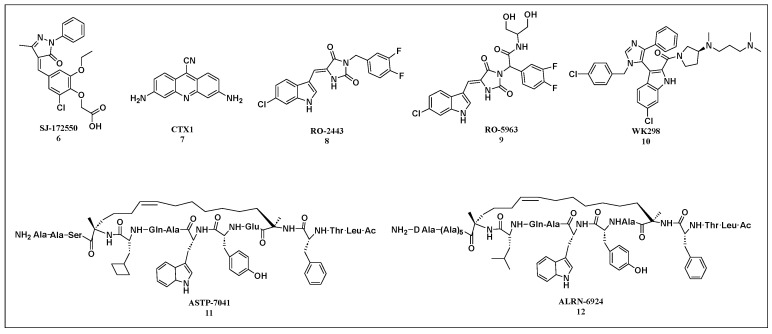
The MdmX inhibitors and Mdm2/MdmX dual inhibitors.

Mdm2 and its homolog MdmX are negative regulators of p53, sharing similar structural domains. Mdm2 inhibitors, designed by mimicking the interaction with key amino acid residues of p53, could, in theory, also inhibit MdmX. However, most existing Mdm2 inhibitors exhibit relatively weak binding affinity for MdmX. Only a few p53-MdmX inhibitors have been reported, including SJ-172550, CTX1, RO-2443, RO-5963, WK298, ATSP-704, and ALRN-6924 (Figure 6).

Small-molecule inhibitors offer several advantages, including high chemical stability, excellent oral bioavailability, and rapid clinical development. The MdmX small-molecule inhibitor SJ-172550 (compound 6) binds to the p53-binding pocket of MdmX, preventing the p53-MdmX interaction. This activation of wtp53 leads to an increase in p21 protein levels and induces apoptosis in MdmX-overexpressing retinoblastoma cells [89,90]. Karan et al. [91] identified CTX1 (compound 7) as an MdmX inhibitor through cellular screening. By competitively disrupting the interaction between p53 and MdmX, CTX1 promotes the expression of p53 and its transcriptional target, p21. This restoration of p53 activity subsequently induces apoptosis in the MCF7 breast cancer cell line.

Through high-throughput screening of the MdmX protein, RO-2443 (compound 8) was identified as one of the first small molecules to bind Mdm2/MdmX with nanomolar affinity. However, its poor solubility limits its suitability for further intracellular evaluation and research. To improve solubility, a more soluble analog, RO-5963 (compound 9), was developed. This compound demonstrated superior anti-cancer activity compared to Nutlin-3a in cancer cells overexpressing MdmX. WK298 (compound 10), the first small molecule successfully co-crystallized with MdmX, occupies the Phe19, Trp23, and Leu26 p53-binding pockets of MdmX, with a binding mode similar to that seen in Mdm2. However, WK298 exhibited relatively weak binding affinity for MdmX [92].

In addition to small-molecule inhibitors, significant progress has been made with MdmX peptide inhibitors. Chang et al. [93] designed a dual Mdm2/MdmX inhibitor, ATSP-7041 (compound 11), which induces p53 target genes in a dose-dependent manner in wtp53 cells. The compound significantly enhances the expression of p21 and Mdm2 mRNA levels. ATSP-7041 demonstrates favorable cellular permeability, in vivo stability, and pharmacokinetic (PK) properties, thereby augmenting its therapeutic efficacy against Mdm2-overexpressing tumors. Stapled peptides have also seen continued development in recent years. Pairawan et al. developed the dual Mdm2/MdmX stapled peptide inhibitor, ALRN-6924 (compound 12), which exhibited high efficacy in several breast cancer cell lines with wtp53 [94]. ALRN-6924 also showed significant anti-tumor activity in acute myeloid leukemia (AML), solid tumors, and lymphoma with wtp53, while maintaining an acceptable safety profile [94].

Currently, there are very few MdmX inhibitors or Mdm2/MdmX dual inhibitors in clinical trials, with ALRN-6924 being the only reported dual inhibitor that has advanced to clinical testing. To translate these promising compounds into clinical applications, it is essential to further optimize and refine the properties of both small-molecule and peptide inhibitors. MdmX small-molecule inhibitors, and Mdm2/MdmX dual small-molecule inhibitors, have become a central focus in current research due to their potential for oral administration. However, challenges related to selectivity still need to be addressed. In contrast, Mdm2/MdmX peptide inhibitors offer significant advantages in terms of targeting precision and low toxicity, though their pharmacokinetic limitations must still be overcome. Future research should combine structural biology, medicinal chemistry, and clinical needs to facilitate the complementary development of both small-molecule and peptide inhibitors, ultimately advancing the clinical translation of Mdm2/MdmX inhibitors.

### 3.3. Other Emerging p53-Based Therapeutic Strategies

In addition to the pharmacological approaches currently under development, gene therapy targeting p53 has emerged as a highly promising strategy for cancer treatment. Recently, gene therapy has seen a resurgence of interest [95,96]. Gendicine, a recombinant human p53 adenovirus developed by Shenzhen SiBiono GeneTech, was approved by the China Food and Drug Administration (CFDA) in 2003 for the treatment of head and neck squamous cell carcinoma (HNSCC). Since its approval, Gendicine has been administered to thousands of patients across China [97,98,99]. Other adenovirus-based *p53* gene therapies, such as Advexin [100] and SCH-58500 [101], have also shown encouraging results in clinical trials [102]. With the development of more sophisticated viral vectors, *p53* gene therapy is expected to become more effective and widely applicable, potentially as part of combination treatment strategies [103].

Nanoparticles have also been investigated as carriers for *p53* gene therapy due to their low immunogenicity, which makes them less susceptible to inhibition by antibodies, thus prolonging their circulation time and minimizing immune-related adverse effects. Notably, advancements in methods for the targeted delivery of gene products to cancer cells have significantly improved the efficiency and specificity of nanoparticles. For example, the selective delivery of wtp53 can enhance the specificity of *p53* gene therapy, facilitating the restoration of p53 expression specifically in cancer cells and significantly augmenting potent anti-cancer effects in vitro and in various xenograft models [104,105,106]. One such example is SGT-53, a cationic liposome developed by SynerGene Therapeutics, which carries DNA encoding wtp53. In a Phase I clinical trial involving 11 patients with various advanced solid tumors, SGT-53 successfully delivered the *TP53* transgene to metastatic sites and demonstrated anti-cancer effects [107]. Furthermore, cationic liposomes coated with anti-transferrin receptor single-chain antibody fragments (scL) have been used to facilitate the delivery of wtp53 (scL-53). This scL-53 nanocomplex targets tumors via the transferrin receptor, which is not expressed on normal cells, thereby enabling more precise tumor-specific delivery [108].

In recent years, strategies utilizing Proteolysis-targeting chimeras (PROTACs) to restore p53 activity have gained significant attention. Adams et al. [109] linked the Mdm2 inhibitor RG7112 with the ligand VH032 of the E3 ubiquitin ligase VHL, creating the PROTAC molecule YX-02-030, which triggers proteasome-mediated degradation of Mdm2. YX-02-030 not only effectively activates wtp53 in cancer cells but also promotes the degradation of p53-deficient and mutp53-expressing cells, particularly in triple-negative breast cancer (TNBC) cells. This molecule induces apoptosis in TNBC patient-derived xenografts and inhibits tumor growth in TNBC xenograft models in mice, without causing toxicity to normal tissues. Kong et al. [110]. employed the SELEX (Systematic Evolution of Ligands by Exponential Enrichment) method to identify single-stranded DNA (ssDNA) that specifically binds to p53-R175H (a mutated form of p53). They then combined this with the PROTAC strategy to develop dp53m, a molecule that induces the ubiquitin-At the forefront of cancer therapy research, significant progress has been made in activating mutp53 and restoring wtp53 function. However, many challenges still need to be overcome. Identifying drugs that can both effectively exert anti-cancer effects and successfully transition to clinical application remains a complex and pressing task.

Preclinical studies have shown that several mutp53 reactivation compounds exhibit anti-cancer activity, but the underlying mechanisms remain largely unclear. For example, once mutp53 is reactivated, does its anti-cancer effect result from apoptosis, cell cycle arrest, or other mechanisms? Furthermore, do p53 reactivation compounds exert different anti-cancer effects in various cell types with distinct p53 mutations? Since p53 mutations are prevalent in many cancers, and different mutations may respond differently to treatment, understanding these differences is crucial for developing targeted therapies. Additionally, there is still limited understanding of how much mutp53 needs to be reactivated to achieve a durable anti-cancer response. In conclusion, while mutp53 reactivation compounds show promise in cancer therapy, further research is needed to deepen our understanding of their mechanisms, the variability of responses across different cell types, and the optimal level of p53 reactivation necessary for sustained therapeutic effects.

Several molecules that activate the p53 pathway have been successfully developed by blocking the interactions between p53 and its primary negative regulators, Mdm2 and MdmX. Most of these molecules are p53-Mdm2-targeting compounds, which have shown some success in clinical trials but have yet to lead to the approval of any drugs. One of the key challenges in developing cancer therapies focused on restoring wtp53 function is the emergence of drug resistance. This resistance can arise from multiple factors, including *TP53* gene mutations induced by Mdm2 inhibitors, which can disrupt p53’s normal function and allow cancer cells to evade the effects of Mdm2 inhibitors. Furthermore, the upregulation of MdmX, an important regulator of p53, can restore its inhibitory effect on p53. These factors can diminish the therapeutic efficacy of Mdm2 inhibitors, significantly reducing their clinical potential. Additionally, when Mdm2/MdmX inhibitors are used, they may regulate p53 in tumor cells but also lead to the abnormal accumulation of wtp53 in normal cells, potentially causing unnecessary cell death and tissue toxicity. This presents a further challenge in developing effective treatment strategies.

To address the resistance problem associated with Mdm2/MdmX inhibitors, many research teams have focused on exploring combination therapies with chemotherapy drugs, immunotherapies, and other agents. The goal is to significantly reduce the required dosage, improve antitumor efficacy, and lower toxicity, thereby reducing the likelihood of cancer cells developing resistance. Some combination therapies have shown synergistic effects in clinical trials, potentially addressing challenges associated with monotherapy by targeting both related and unrelated mechanisms. This combination strategy offers new insights for overcoming resistance to Mdm2/MdmX inhibitors.

As a key tumor suppressor, p53 plays a critical role in apoptosis, DNA repair, cell cycle regulation, and other cellular processes. Its activation holds great potential for breakthroughs in cancer treatment. While no inhibitors have been approved for clinical use thus far, the potential of p53 activation as a therapeutic strategy for cancer remains highly promising and offers hope for the future.

## Figures and Tables

**Table 1:** Mdm2 inhibitors that have already undergone Phase I clinical trials.

Compounds	Structure	Combination	Object	Phase	Result
RG7112	imidazoline scaffold	Doxorubicin	Soft tissue sarcoma	Phase I NCT01605526	Neutropenia (60%) and thrombocytopenia (45%) were observed [52].
RG7112	imidazoline scaffold		Late-stage solid tumors	Phase I NCT01164033	High-dose treatment for 3 to 5 days was more effective than prolonged weekly or lower-dose daily treatments [53].
AMG232	Piperidone class	Trametinib	Relapsed/Refractory AML	Phase I NCT02016729	The single-dose treatment was 360 mg, and 60 mg when combined with trametinib. Among the 13 patients, 4 (31%) showed a response to the treatment [54].
Milademetan(RAIN-32, DS-3032b)	Spirooxindole		Late-stage solid tumors and lymphomas	Phase I NCT01877382	The maximum tolerated dose was 160 mg in the once-daily 21/28 schedule and 260 mg in the every day 3/14 × 2 schedule (1 cycle was 28 days) [55]
NVP-CGM097 Series	Dihydro-isoquinolinones		Late-stage solid tumor	Phase I NCT01760525	The drug was administered at doses of 10–400 mg weekly for 3 weeks, or 300–700 mg weekly for 2 weeks with a 1-week interval. The maximum tolerated dose was not reached. A portion (39%) of patients responded to the treatment, including one case of partial response and 19 patients with stable disease [56].
HDM201	Pyrrolidono-imidazole		Late-stage solid tumors and hematological wtp53 tumors	Phase I NCT02143635	Among patients with solid tumors, the response rate was 10.3%, whereas in patients with acute myeloid leukemia, the response rates varied across different regimens, being 4.2%, 20%, and 22.2%, respectively (NCT02143635).
Idasanutlin (RO5503781, RG-7388)	Pyrrolidine	Cytarabine	wtp53 cancer patients	Phase I NCT01773408	The remission rate for monotherapy was 18.9%, whereas the remission rate for combination therapy was 35.6% [57].
Idasanutlin (RO5503781, RG-7388)	Pyrrolidine		tumor	Phase I NCT01462175	The drug was administered at a daily dose of 500 mg for 5 days in a 28-day cycle and showed hematological toxicity [58].
RO6839921 (RG7775)	Polyethylene glycolation of pyrrolidine		Solid tumor and AML	Phase I NCT02098967	Among solid tumor patients, 34% had stable disease, whereas the disease control rate for AML patients was 42% [59].
APG-115	Spirooxindole		Advanced solid tumors or lymphomas.	Phase I NCT02935907	The dosing regimen for APG-115 in a 28-day cycle involved administering 100 mg every other day for 21 days [60].
SAR405838 (MI-77301)	pirooxindole		Late-stage solid tumors	Phase I NCT01636479	Each treatment involved 300 mg once daily and was associated with elevated plasma macrophage inhibitory cytokine-1 (MIC-1). 56% of patients experienced stable disease, and 32% remained progression-free at 3 months [60].

## Data Availability

Not applicable.
